# Outcome of capacity building intervention for malaria vector surveillance, control and research in Nigerian higher institutions

**DOI:** 10.1186/s12936-018-2344-z

**Published:** 2018-05-15

**Authors:** Adedayo O. Oduola, Abiodun Obembe, Olukayode J. Adelaja, Adeniyi K. Adeneye, Joel Akilah, Taiwo S. Awolola

**Affiliations:** 1 Vector Biology and Control Research Group, Department of Zoology, University of Ilorin, Ilorin, Nigeria; 2 Department of Biotechnology, Kwara State University, Malete, Nigeria; 3 Molecular Entomology and Vector Control Research Laboratory, Public Health and Epidemiology Division, Nigerian Institute of Medical Research, Lagos, Nigeria; 4 Integrated Vector Management Branch, National Malaria Elimination Programme, Abuja, Nigeria

**Keywords:** Capacity building intervention, Training, Malaria, Vector control, Personnel, Research, Surveillance, Nigerian institutions

## Abstract

**Background:**

Despite the availability of effective malaria vector control intervention tools, implementation of control programmes in Nigeria is challenged by inadequate entomological surveillance data. This study was designed to assess and build the existing capacity for malaria vector surveillance, control and research (MVSC&R) in Nigerian institutions.

**Methods:**

Application call to select qualified candidates for the capacity building (CB) intervention training programme was advertised in a widely read newspaper and online platforms of national and international professional bodies. Two trainings were organized to train selected applicants on field activities, laboratory tools and techniques relevant to malaria vector surveillance and control research. A semi-structured questionnaire was administered to collect data on socio-demographic characteristics of participants, knowledge and access of participants to field and laboratory techniques in MVSC&R. Similarly, pre and post-intervention tests were conducted to assess the performance and improvement in knowledge of the participants. Mentoring activities to sustain CB activities after the training were also carried out.

**Results:**

A total of 23 suitable applicants were shortlisted out of the 89 applications received. The South West, South East and North Central geopolitical zones of the country had the highest applications and the highest selected number of qualified applicants compared to the South South and North East geopolitical zones. The distribution with respect to gender indicated that males (72.7%) were more than females (27.3%). Mean score of participants’ knowledge of field techniques was 27.8 (± 10.8) before training and 67.7 (± 9.8) after the training. Similarly, participants’ knowledge on laboratory techniques also improved from 37.4 (± 5.6) to 77.2 (± 10.8). The difference in the mean scores at pre and post-test was statistically significant (p < 0.05). Access of participants to laboratory and field tools used in MVSC&R was generally low with insecticide susceptibility bioassays and pyrethrum spray collection methods being the most significant (p < 0.05).

**Conclusions:**

The capacity available for vector control research and surveillance at institutional level in Nigeria is weak and require further strengthening. Increased training and access of personnel to relevant tools for MVSC&R is required in higher institutions in the six geopolitical zones of the country.

**Electronic supplementary material:**

The online version of this article (10.1186/s12936-018-2344-z) contains supplementary material, which is available to authorized users.

## Background

Malaria is a major public health problem in most parts of the world. Approximately 216 million people contracted the disease in 2016 resulting in an estimated 445,000 deaths, with the WHO African Region accounting for 91% of all these deaths [1]. Nigeria and The Democratic Republic of Congo account for over 37% of the estimated total cases of malaria deaths globally [1].

In Nigeria, the disease accounts for about 60% of outpatient visits, 30% of hospitalizations, 10% of low birth weight and 11% of maternal mortality in Nigeria [2]. The use of long-lasting insecticidal nets (LLINs) and indoor residual spraying (IRS) are two strategies under the integrated vector management (IVM) for malaria control. While there have been several mass campaign to distribute and promote the use of LLIN, IRS remains very limited because of high costs. These strategies are also known to have a community-wide effect; thus persons who do not receive personal protection can still benefit from these interventions [3].

In spite of the high coverage campaign and accessibility associated with LLINs, only very few states had adopted and implemented IRS as a major component of the malaria control programme. In the few states where IRS had been adopted, re-spraying implementation remains limited and the utilization of other interventions, such as larval source management, remains marginal. Where these strategies are deployed, the research capacity for monitoring insecticide resistance and transmission dynamics and vector control activities in most of the local control programmes remain very limited and unsustainable. In most cases, implementation of vector control programmes have always preceded a careful systematic and continuous monitoring and surveillance of target species, thus putting control programmes at risk of failure [4]. The unavailability of malaria entomological data to guide implementation and monitoring of vector control strategies is often attributed to weak capacities in local control programmes. Existing literatures have clearly shown that over 100 million nets across have been distributed in the last 8 years in Nigeria [2]. Despite this, only very few control programmes have been strengthened to monitor the impact of these interventions on vector population, behaviour and continued susceptibility to insecticides approved for malaria vector control.

Okorie et al. [4] have shown that the proportion of malaria entomological studies carried out between year 2000 and 2010 was lowest in the North East followed by North West, South South, South East and the North Central geo-political zones of Nigeria. The South West geopolitical zone was observed to have an extensive data set on malaria entomology within the same time frame. The reason behind this has not been investigated. However, interaction with managers at the National Malaria Elimination Programme shows that the very few scientists available and capable of sustaining vector control programmes are located in research institutes or the universities (Oduola pers. comm.). However, there exists a major gap between research and programme implementation. This assertion is buttressed by the availability of research information that has not been designed to meet the operational health needs of state malaria control programmes. It is based on this background that this study was designed to assess and build the capacities of eligible personnel identified from institutions in each of the six geopolitical zones in Nigeria. This group of personnel will form a critical mass that will improve the generation of malaria vector control research data capable of guiding the implementation of interventions that can support local vector control activities and reduce malaria burden.

## Methods

### Recruitment of participants for capacity building workshop

To ensure that participants from the universities and allied research institutions in all the six geopolitical zones in Nigeria have equal opportunity to apply, the call for application for the training programme was advertised in a national daily with a widespread readership and coverage. Other website links such as The Research Academia, Research Gate and social network platforms of societies such as Entomological Society of Nigeria and Parasitology and Public Health Society of Nigeria were used for the dissemination of the training workshop.

In order to select qualified candidates, previous malaria vector control research activities of the candidates formed part of the criteria set out for the selection. Applicants were also requested to submit their curriculum vitae, nomination letter from their employers and a one-page motivation letter indicating the relevance of the training programme to their organization and job responsibilities.

### Description of training

Two trainings were organized. The first training was held between 24th October through 1st November, 2016 at the Department of Zoology University of Ilorin, Nigeria. The second part of the training which focused mainly on exposing participants to laboratory tools and techniques relevant to MVSC&R was organized on 27th February through 3rd March, 2017 at the Molecular Entomology Laboratory of the Nigerian Institute of Medical Research, Lagos.

### Adaptation and review of training materials

The training manuals required for each session of the workshop were identified ahead of the training based on need assessment carried out from reported literatures and suggestions picked from the application of the participants. The training manuals and protocols were prepared ahead of the training and provided to the trainees during the workshop. The standard morphological identification keys [5, 6] and training manual on malaria entomology [7] were part of materials used during the training. Soft copies of all the manuals and prepared PowerPoint slides were also submitted by trainers were made available to the trainees in Compact Disc (CD) storage devices.

### Selection of training faculties

Resource persons invited for this training were experienced trainers and consultants from the following field: Malaria, Sociology, Vector Ecology and Private Partners in Vector Control, Entomology, Parasitology and Molecular Biology. They are also very skilled in the use of participatory techniques to facilitate knowledge and skills acquisition.

### Collection of data and assessment of improvement in knowledge after implementation

Two types of assessments were conducted during the training. The first assessment was the pre- and post-intervention tests to evaluate the impact of the training on the performance and improvement in knowledge of the participants. The second assessment was a self-administered questionnaire to assess the participants’ socio-demographic data and evaluation of the capacity building workshop. This questionnaire also assessed the participants’ previous access to techniques and tools in MVSC&R.

### Mentoring activities on mobile applications

In order to sustain the institutional capacity building and enhance the technical skills of trained participants to carry out relevant studies in MVSC&R, a follow-up and mentoring process was initiated to guide the trainees in this process. A “WhatsApp” group platform was created to facilitate interaction between the participants and trainers designated as mentors after the training. The choice of the WhatsApp social network platform was because of its ease of use and acceptability among the trainees. This paved way for real time sharing of information on relevant issues and research activities after the workshop. Mentors were expected to provide technical advice and support to the trainees in the design and implementation of their individual vector control research plan. Mentors were also expected to provide progress report on the compliance of the mentees to checklist of activities. Five activities were earmarked for the follow-up mentoring processes. The checklist of activities included: development of concept notes, development of a surveillance proposal that fits into the national malaria surveillance gathering plans, formation of research group in their institutions/localities, stepping-down of training among group members, visiting respective state malaria programme offices to align with vector surveillance initiatives of the national malaria elimination programme. The improvement in knowledge from the pre and post-tests including the compliance of the participants to the checklist of activities formed the major yardstick for assessing the impact of the capacity building intervention.

### Data analysis

Data obtained from recruitment of trainee exercise, pre- and post-test questionnaires and the administered semi-structured questionnaires were coded and analysed to generate descriptive frequencies and charts using SPSS version 20 software. Paired sample t test was used to compare the significance (5% level of significance) in the mean score of the participants before and after the CB training intervention. Chi Square test was also used to compare the participants’ knowledge of field, laboratory tools and techniques in malaria vector surveillance and research and control.

## Results

### Distribution of applicants for the capacity building intervention programme

**Fig. 1:**
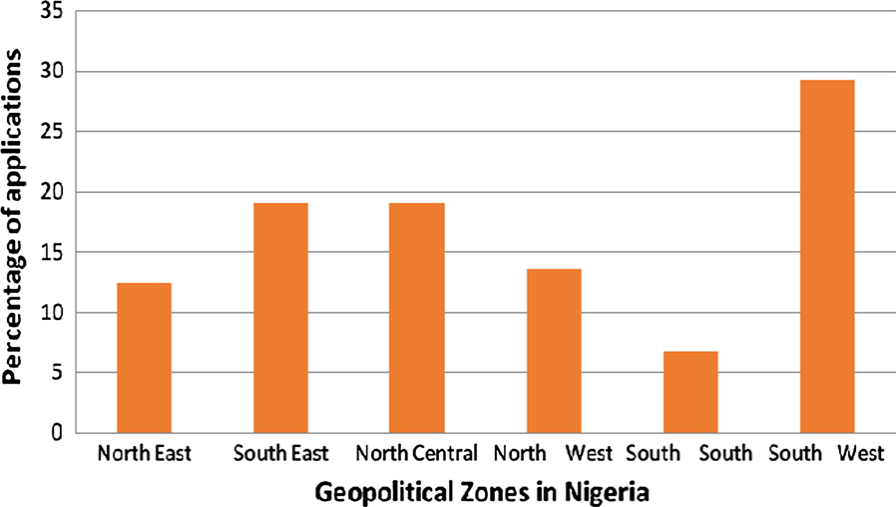
Number of applications received from each of the six geo-political zones in Nigeria

A total of 89 applications were received from the six geo-political zones in Nigeria (Fig. 1). The distribution of the applicants (Fig. 1) showed that the highest number 26 (29.2%) of applications were received from the South West geopolitical zone compared to the South South, where only 6 (6.7%) applications were received. Other applications received were from the South East 17 (19.1%), North Central 17 (19.1%), North East 11 (12.4%), and North West 12 (13.5%) region (Fig. 1). Twenty-four (27.0%) of the 89 applicants who met the criteria were later shortlisted for the training workshop.

### Socio-demographic profile of the participants selected for the capacity building training workshop

**Fig. 2:**
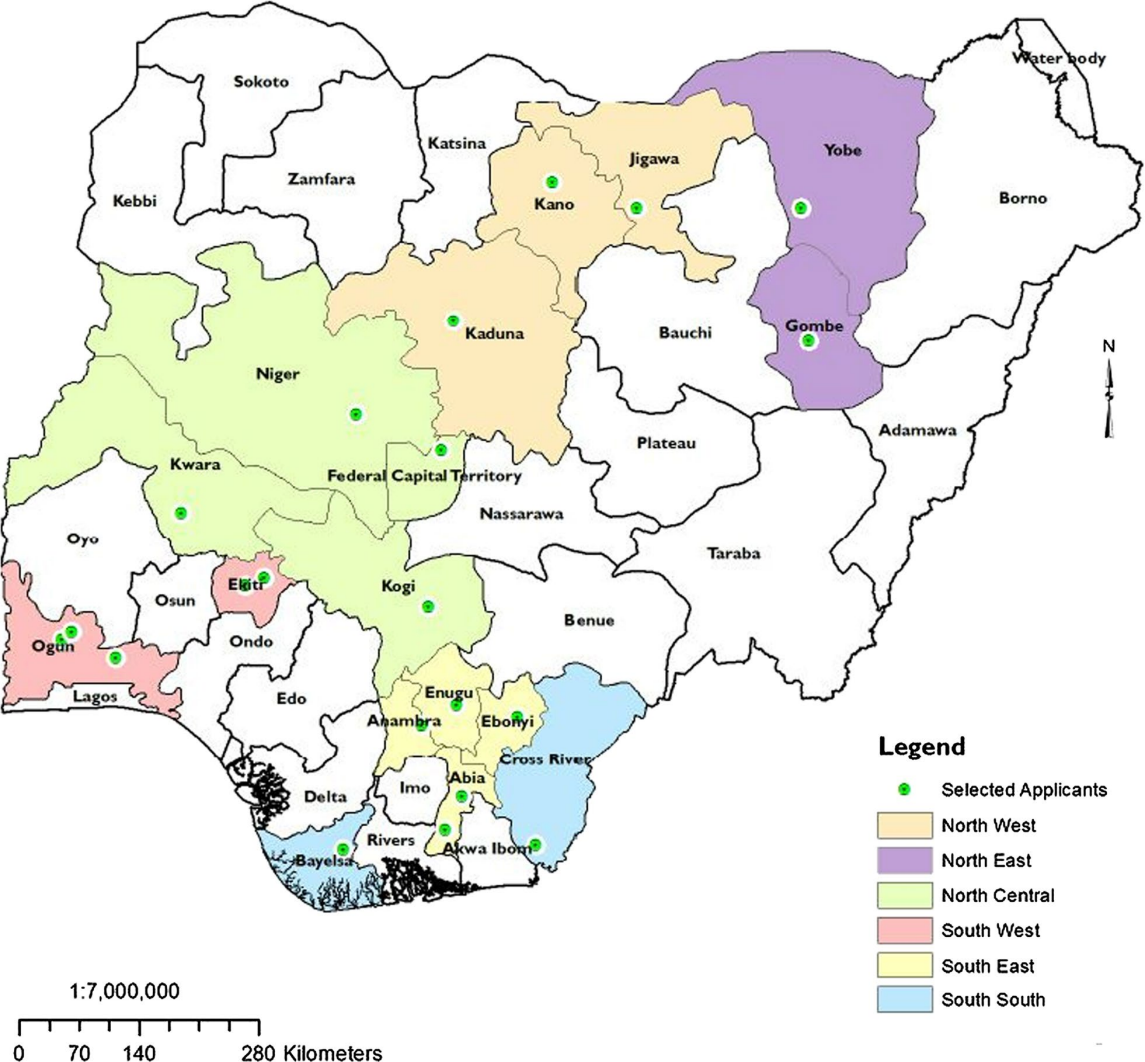
Distribution of participants for the CB training workshop across the six geopolitical zones in Nigeria

The participants for the workshop were drawn from the six geopolitical zones in Nigeria (Fig. 2). A total of 24 qualified applicants were selected for the first phase of the CB workshop, of which only 23 applicants from 22 tertiary institutions in Nigeria attended (Table 1). The distribution of the selected applicants and geopolitical zones has been described (Table 2). The age stratification of the trainees indicated that 13 (56.5%) were between age 26–35 years while 10 (43.5%) were between 36 and 45 years. Only 8 (31.8%) were still single as at the time of study and 15 (65.2%) were married (Table 2). The distributions with respect to level of education showed that all (100.0%) the participants had tertiary education and 13 (56.5%) were at their early career stage. The latter category were people with M.Sc., Ph.Ds, Assistant Lecturers currently undergoing their Ph.D. studies while the middle career stage had frequency of 9 (39.1%). Those under this category were academia at Lecturer II/I designation in their respective institutions. However, only one (4.5%) of the participants fell under the senior level career stage (Table 2). One of the participants dropped out of the training before the final evaluation and post-test exercise was conducted.

**Table 1:** Gender, geopolitical zone and institution spread of applicants selected for the capacity building training workshop

S/N	Region	Address/institution	Sex
1	North Central	Department of Biological Sciences, Federal University of Technology, Minna	M
2	North Central	Department of Parasitology and Entomology, Faculty of Veterinary Medicine, University of Abuja, Abuja-FCT, Nigeria	M
3	North Central	Department of Biological Sciences, Kogi state University, Anyigba	F
4	North Central	Department of Biological Sciences, University of Agriculture, Makurdi, Benue State	M
5	North West	Department of Biological Sciences, Kaduna State University	M
6	North West	Department of Biological Sciences, Sule Lamido University, Kafin Hausa, Jigawa State	M
7	North West	Department of Biological Sciences, Federal University, Dutse, Jigawa State	M
8	North East	Department of Biological Sciences, Gombe State University	M
9	North East	School of Science Education, Federal College of Education (TECH), Potiskum, Yobe	M
10	North East	Department of Biological Sciences, University of Maiduguri	M
11	North East	Department of Biological Sciences, Gombe State University	M
12	South West	Department of Pure and Applied Zoology, Federal University of Agriculture, Abeokuta	F
13	South West	Federal University, Oye-ekiti	F
14	South West	Olabisi Onabanjo University, Ago Iwoye, Ogun state	M
15	South West	Department of Pure and Applied Zoology, Federal University of Agriculture, Abeokuta	M
16	South West	Department of Zoology, Ekiti State University, Ado-Ekiti	M
17	South South	Calabar Institute of Tropical Disease, Research and Prevention, University of Calabar Teaching Hospital	M
18	South South	Isaac Jasper Boro College of Education, Sagbama, Yenagoa, Bayelsa	M
19	South East	Department of Zoology and Environmental Biology, University of Agriculture Umudike, Abia	M
20	South East	Applied Biology Unit, Department of Biological Sciences, Ebonyi State University	F
21	South East	Department of Parasitology and Entomology, Nnamdi Azikiwe University, Awka, Anambra	F
22	South East	Biology/Microbiology Department, Abia State Polytechnic, Abia	F
23	South East	National Arbovirus and Vectors Research Center, Enugu	M

**Table 2:** Socio-demographic data of participants at the malaria capacity building workshop

Parameters	Distribution of participants
	Frequency (%)
Total no. of participants	23
Geopolitical zones	
South-West	5 (21.7)
South-East	4 (17.4)
South-South	2 (8.7)
North-Central	5 (21.7)
North-West	3 (13.0)
North-East	4 (17.4)
Ethnicity	
Igbo	6 (26.1)
Hausa	3 (13.0)
Yoruba	6 (26.1)
Minority	8 (34.8)
Gender	
Male	17 (73.9)
Female	6 (26.1)
Age group (years)	
26–35	13 (56.5)
36–45	10 (43.5)
Designation (career status)	
Early career	13 (56.5)
Middle career	9 (39.1)
Senior career	1 (4.4)

### Contents and description of training

Two trainings were organized. The first training was held at the Department of Zoology University of Ilorin, Nigeria. The training was in three phases: the theoretical sessions; field sessions; and laboratory sessions. The field visits demonstrated Anopheles ecology, sampling of mosquitoes using different techniques including Pyrethrum Spray Collection (PSC), mounting of Exit Traps and usage of Centers for Disease Control (CDC) Light traps. Techniques taught also included transportation of live mosquitoes and preservation and morphological identification of mosquitoes. The laboratory aspect included sessions on insecticide susceptibility testing, larval bioassays recording, plotting the time mortality charts and interpretation of results. Other issues in malaria vector control such as operation research, ethical issues, social advocacy and community mobilization were also treated. Twenty-three participants attended the training at the University of Ilorin.

The second part of the CB workshop focused mainly on exposing the participants to laboratory tools and techniques relevant to malaria vector control research was organized at one of the collaborating center, the Molecular Entomology Laboratory at the Nigerian Institute of Medical Research Yaba Lagos. The training included DNA extraction techniques, polymerase chain reaction (PCR) assays, electrophoresis, photo-documentation and enzyme linked immunosorbent assays (ELISA) for determining sporozoite infection rates and blood meal source analysis. The participants were also exposed to the principles and practices of insect rearing at a standard insectary facility at the collaborating centre. Twelve participants whose research proposals submitted after the first training required laboratory skills.

### Discipline and affiliations of training faculties

A total of 17 facilitators who are professionals from different fields relevant to the CB objectives of the training workshop were invited for the training. The institution and disciplines of the facilitators is described in Table 3.

**Table 3:** Discipline and institution of faculties selected for the CB workshop

S/N	Institution	Discipline
1	University of Ilorin Teaching Hospital, Ilorin	Malariologist
2	University of Ilorin	Insect Ecologist
3	University of Ilorin	Parasitologist
4	United States Presidential Malaria Initiative. African Indoor Residual Spray	Technical Manager (Indoor Residual Spraying)
5	Integrated Vector Management National Malaria Elimination Programme, Abuja	IVM, Federal Ministry of Health, Policy
6	World Health Organization	National Programme Officer (Malaria)
7	Nigerian Institute of Medical Research, Lagos	Malaria Entomologist
8	Nigerian Institute of Medical Research, Lagos	Medical Sociologist
9	University of Ilorin	Molecular Biologist
10	Federal University Oye, Ekiti State	Pesticide Scientist
11	Lead City University, Ibadan	Entomologist
12	University of Ilorin	Entomologist/Field Officer
13	Harvestfield Industries Ltd	Indoor Residual Spray Specialist
14	Kwara State University	Field Entomologist
15	Osun State Malaria Elimination Programme	Public Health Specialist/Training Manager
16	University of Ilorin	Social Network Manager
17	Nigerian Institute of Medical Research, Yaba, Lagos	Biochemist

### Participants’ knowledge of field techniques in MVSC&R before and after training

**Fig. 3:**
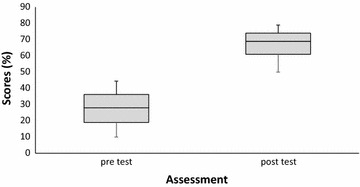
Minimum, percentiles and maximum scores of participants before and after training in field techniques

In the pre-test, only three (13.0%) of the 23 persons scored 40% and above. In contrast, all but one participant who did not write the post-test successfully scored 50% and above (Table 4). The minimum and maximum score of participants before the training were 10 and 47 respectively with mean score of 27.8 (± 10.8). Following the post-test, the minimum and maximum score was 50 and 83 respectively and mean was 67.7 (± 9.8) (Fig. 3). The difference in the mean scores at pre and post-test was statistically significant (p < 0.001; t = − 17.02). Overall average shows 143.5% increase in knowledge (Additional file 1).

**Table 4:** Distribution of scores of participants during the pre-and post-test assessments of knowledge on field techniques

	Pre-test number of participants	Participants score range (%)	Post-test number of participants
	8	10–20	0
	5	21–30	0
	7	31–40	0
	3	41–50	0
	0	50–60	5
	0	61–70	7
	0	> 71	10
Total	23		22

### Participants’ knowledge of laboratory techniques in MVSC&R before and after training

**Fig. 4:**
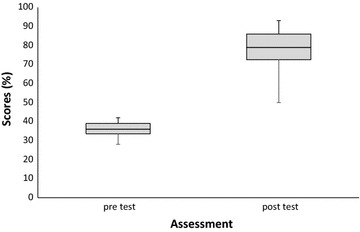
Minimum, percentiles and max scores of participants before and after training in laboratory techniques

All the 12 trainees selected for the 2nd capacity building training workshop participated in both the pre- and post-test evaluation exercises. In the pre-test, only four (33.3%) of the 12 persons scored 40% and above. In contrast, all the participants successfully scored 50% and above in the post-test (Table 5). The pre-assessment on knowledge of malaria vector surveillance and laboratory research techniques before the training revealed a minimum and maximum score of 28 and 48 respectively with overall average of 37.4 (± 5.6). Following the post-test, the minimum and maximum score was 50 and 89 respectively and mean was 77.2 (± 10.8) (Fig. 4). The difference in the mean scores at pre- and post-test was statistically significant (p < 0.001; t = − 10.82). The overall average post-test score shows 106.4% increase in knowledge acquired by participants at the training workshop compared to their average pre-test score (Additional file 2).

**Table 5:** Distribution of scores of participants during the pre-and post-test assessments of knowledge on laboratory techniques

	Number of participants (pre-test)	Participants score range (%)	Number of participants (post-test)
	1	20–29	0
	7	30–39	0
	4	40–49	0
	0	50–59	1
	0	60–69	1
	0	70–79	5
	0	> 80	5
Total	12		12

### Evaluation of participants’ previous entomological experience

The results of evaluation carried out to determine the previous access of participants to techniques and tools in MVSC&R is shown in Table 6. None of the participants from all the six geopolitical zones across the country were familiar with exit traps (Table 6). Only one individual each (33.3%) from North-East, South-East and South-South had previous engagement with the CDC light trap. This amounted to only 3 (13.6%) of all the participants from all the 22 tertiary institutions who had used the CDC light traps before this training (Table 6). Previous involvement with questionnaire designs and administration before the workshop showed that 18 (82%) of the participants spread across each of the six geopolitical zones had previously been involved. Assessment of involvement with PSC also showed that 7 (31.8%) participants from all the geopolitical zones except South West and North West had previous experience with PSC. The number of participants with prior experience with PSC was significantly higher (p = 0.044) compared with those without. Only two (100%) individuals from North East were acquainted with IRS. There was no significant difference (p = 0.878) in the number of participants 8 (36.4%) who had previous involvement with insecticide susceptibility bioassays compared to those who had not. All the geopolitical zones except the North West had at least one individual each having previous experience with larval bioassays. The number of participants having previous involvement with programme implementation involving communities was higher in all the six geopolitical zones.

**Table 6:** Evaluation of participants’ previous access to field tools and techniques in malaria vector surveillance control and research

Parameters	Participants experience with respect to geopolitical zones n (%)							p value
	North-Central	North-East	North-West	South-West	South-East	South-South	Total	
Previous involvement with exit light traps								^a^
Yes	0	0	0	0	0	0	0	
No	5 (22.7)	4 (18.2)	3 (13.6)	4 (18.2)	4 (18.2)	2 (9.1)	22 (100)	
Previous involvement with CDC light traps								0.414
Yes	0	1 (33.3)	0	0	1 (33.3)	1 (33.3)	3 (13.6)	
No	5 (26.3)	3 (15.8)	3 (15.8)	4 (21.1)	3 (15.8)	1 (5.3)	19 (86.3)	
Previous involvement with questionnaire								0.841
Yes	4 (22.2)	3 (16.7)	2 (11.1)	4 (22.2)	3 (16.7)	2 (11.1)	18 (81.8)	
No	1 (25.0)	1 (25.0)	1 (25.0)	0	1 (25.0)	0	4 (18.2)	
Previous involvement with PSC								0.044
Yes	1 (14.3)	3 (42.9)	0	0	1 (14.3)	2 (28.6)	7 (31.8)	
No	4 (26.7)	1 (6.7)	3 (20.0)	4 (26.7)	3 (20.0)	0	15 (68.2)	
Previous involvement with mosquito identification								0.220
Yes	1 (10.0)	3 (30.0)	2 (20.0)	1 (10.0)	1 (10.0)	2 (20.0)	10 (45.5)	
No	4 (33.3)	1 (8.3)	1 (8.3)	3 (25.0)	3 (25.0)	0	12 (54.5)	
Previous involvement with mosquito identification keys								0.642
Yes	2 (16.7)	3 (25.0)	2 (16.7)	1 (8.3)	3 (25.0)	1 (8.3)	12 (54.5)	
No	3 (30.0)	1 (10.0)	1 (10.0)	3 (30.0)	1 (10.0)	1 (10.0)	10 (45.5)	
Previous involvement with IRS								0.078
Yes	0	2 (100)	0	0	0	0	2 (9.1)	
No	5 (25.0)	2 (10.0)	3 (15.0)	4 (20.0)	4 (20.0)	2 (10.0)	20 (90.1)	
Previous involvement with susceptibility bioassays								0.027
Yes	0	4 (50.0)	1 (12.5)	0	2 (25.0)	1 (12.5)	8 (36.4)	
No	5 (35.7)	0	2 (14.3)	4 (28.6)	2 (14.3)	1 (7.1)	14 (63.6)	
Previous involvement with larval bioassays								0.878
Yes	1 (20.0)	1 (20.0)	0	1 (20.0)	1 (20.0)	1 (20.0)	5 (22.7)	
No	4 (23.5)	3 (17.6)	3 (17.6)	3 (17.6)	3 (17.6)	1 (5.9)	17 (77.3)	
Previous involvement with community project								0.492
Yes	3 (16.7)	3 (16.7)	2 (11.1)	4 (22.2)	4 (22.2)	2 (11.1)	18 (81.8)	
No	2 (50.0)	1 (25.0)	1 (25.0)	0	0	0	4 (18.2)	

^a^ No measure of association because no values were recorded for ‘yes’ variable

### Evaluation of participants’ previous access to laboratory tools in MVSC&R

An assessment carried out to determine the previous access of participants to tools required for vector control research showed that all the participants have never carried out a polymerase chain reaction assay, prepared an agarose gel, used an ultraviolet photo-documentation system, and designed a primer for polymerase chain reactions before. When prompted if they had ever extracted DNA, determined sporozoite infection rate or ever used a spectrophotometer microplate reader before, only 1 (8.3%) of the participants had performed each of these three tasks before. When asked about the possession of a functional insectary in the respective institutions, only 2 (16.7%) of the participants had insectary in their institutions (Table 7).

**Table 7:** Assessment of participants’ access to laboratory tools in malaria vector surveillance and control research

Parameters	N (%)
Have you ever carried out a polymerase chain reaction assay before?	12 (100)
Have you ever been involved in DNA extraction before?	11 (91.6)
Have you designed or used a primer before?	12 (100)
Have you ever prepared an agarose gel before?	12 (66.7)
Have you ever used a ultra violet photo-documentation system before?	12 (66.7)
Have you ever determined sporozoite infection rate before?	11 (91.7)
Have you ever used a spectrophotometer/microplate reader before?	11 (91.6)
Do you have an insectary in your institution?	10 (83.3)

*N* number of participants with negative response

### Compliance of trainees to the mentoring checklist of activities

**Fig. 5:**
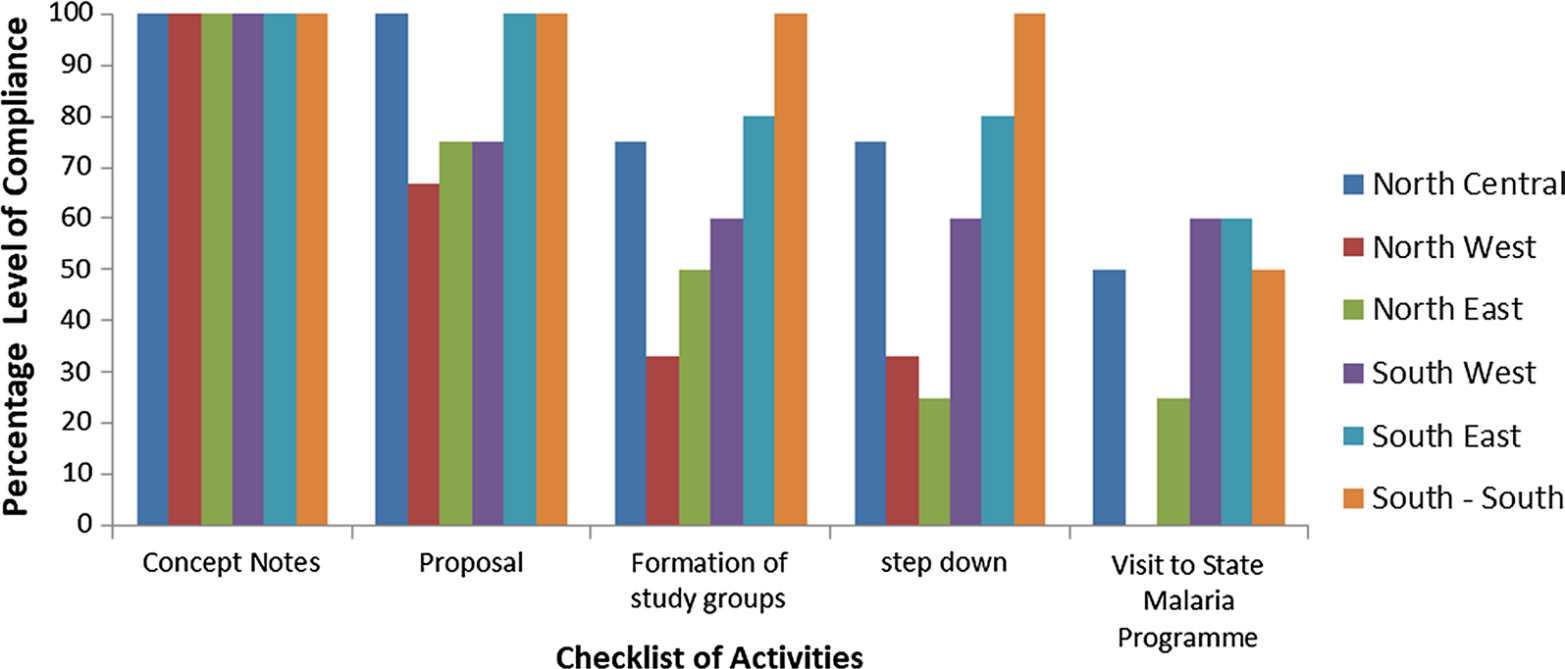
Compliance of participants to mentoring checklist of activities

All 23 participants submitted concept notes describing the research activities they intended to use to cascade knowledge in their home institutions (Fig. 3). Of these, 18 (83%) participants developed a proposal and 16 (65.2%) formed their research groups (Table 6). Fourteen (60.1%) of the participants stepped down the training following which only 6 (26.1%) visited the local Malaria Elimination Programme in their states to align with the surveillance efforts (Fig. 5).

## Discussion

Implementing sustainable malaria control programmes require strong institutions, funding and a critical mass of skilled personnel to drive the well-proven vector control strategies. A good operational research component is also required to generate data capable of bridging health need gaps and the formulation of policy capable of reducing malaria burden. Identifying the right personnel and institution with focus on the field of specialization is particularly important in building sustainable national vector control and surveillance programmes. The distribution of applicants across all six geopolitical zones in Nigeria emphasizes the need of empowering scientists in these regions to consistently generate this body of knowledge, which is a precursor to implementing effective vector control programmes. Higher number of applications received from the South Western geopolitical zones of Nigeria when compared to the other regions confirmed the earlier speculations of existing active facilities involved in malaria vector monitoring and surveillance in South West Nigeria. According to Okorie et al. [4] the South-West geopolitical zone was observed to have an extensive data set on malaria entomology when compared to other regions considered within the same time frame. This simply confirmed the presence of active entomologists [8] and may have stimulated the higher training-seeking behaviour of researchers from this region.

While the South East, South West, North Central and North East had suitable number of qualified applicants to make up the four training slots allocated to each of the six geopolitical zones, this was not the same for the South South and North West geopolitical zones. This necessitated the consideration of some additional applicants from zones that had fully utilized their slots. This outcome may likely be an indication of an existing weak research infrastructures and a dearth of professionals available for malaria vector control research. In order to avoid labour crisis [9] or in availability of professionals to carry out interventions, such as vector monitoring and surveillance, there is the need for stakeholders which includes funders, training institutions and the NMEP to consider training and equipping a pool of competent scientists that will be able to support local research needs and capacity for a sustainable malaria vector control and surveillance programmes in each of the six geopolitical zones in Nigeria. According to Uneke et al. [9] short falls in the supply of professional personnel should be prioritized. Training of personnel should be prioritized and not be left to chance or be driven by disease incidences as it is reported to be done in most low medium income countries [10].

The outcome of this capacity building intervention has provided information on existing gap in the access of scientists or practicing Medical Entomologists in Nigeria to relevant skills required to engage in malaria vector surveillance information gathering. According to Beaglehole [11] low number of competent researchers is a major problem facing research and development in Nigeria. Nigeria and Ghana have been featured as countries with the lowest researchers per million [12].

The non-familiarity of participants selected from 22 higher institutions in Nigeria with relevant and important tools, skills and techniques indicate a wide gap between knowledge and practice of Medical Entomology in Nigerian Institutions. The majority of the participants in this study were not familiar with important laboratory and field tools to carry out vector borne disease surveillance. This requires a conscious implementation of a sustainable training programme to update the knowledge of personnel in this field. Another way out would be the upgrading of current curriculum of selected tertiary institutions to meet the demands and challenges of recent trends in vector borne diseases. It is evident that there is either the absence of tools or skilled personnel to communicate this knowledge to students in training. The age (26–45) distribution of the participants from the 22 institutions across six geopolitical zones in Nigeria who attended the CBP also attested to this. The distribution also showed that majority of these participants (54.5%) and (40.9%) were at their earlier and middle career stage respectively. The non-familiarity of majority of these participants with vector surveillance, control and research tools is worrisome suggesting that they may not have been exposed to this during their tertiary or university training. These categories of personnel without exposure to these tools were likely to have passed through several institutions within the country in the last 5–20 years (considering the age group of the participants). These graduates without this training are in the helms of vector control, research, monitoring. They are also likely to be involved in the training of vector biologists that are to support future local control programmes. The assessment of participants’ previous entomological experience was used to conclude that the capacity available for vector control research at institutional level in Nigeria is weak. Strengthening research, teaching and training in Nigerian institutions will increase the required generation of a critical mass of well-trained scientists that can support local malaria vector control and research programmes. Control programmes that require these skills will have to look for an avenue of organizing additional training to meet these needs.

The significant improvement in knowledge of the participants after the capacity building training intervention laid emphasis on existing dearth in knowledge of potential pool of skilled workforce that can be enlisted to support vector surveillance and research activities in Nigeria. This present gap justifies the need to implement further CB training in the management of knowledge in vector control, surveillance and research. The knowledge acquired will improve the practice and productivity of the trained personnel for effective malaria vector control research and surveillance activities.

Establishment of improvement in knowledge after training is not sufficient in evaluating the impact of a CB training programme. As such, a checklist of activities or mutually agreed goals was designed to develop participants on specific areas of competencies [13]. Experienced researchers in relevant field of malaria research were assigned as mentors to follow up on the mentees, providing assistance to ensure that they comply with series of activities which will allow them build upon the needs and knowledge gaps identified during the training [14]. Mentors assigned to each of the trainee provided information on the compliance of the participants from each of the geopolitical zones. According to Strauss et al. [15], the goal of assigning mentors was to retain faculty that will be productive. Productivity here may be the generation of data that can support local malaria vector surveillance and control programme or implementation of malaria control activities. Mentoring has been described as a way by which well-experienced professionals can connect with less-experienced or junior colleagues in order to offer guidance and assistance in their career progress [16, 17]. The initial response from the first activities earmarked for the short-term assessment was encouraging and had a higher degree of compliance among the participants. The dwindling trend in compliance with the checklist of activities by the participants indicated a major weakness and flaw to drive conceptual ideas at the academic level to the community for implementation. The absence of funds and inability of trainees to access funds to support the implementation of their proposal at the latter stage became an impediment and limitation to the mentoring aspect of this CB programme. As such, capacity building training organizers in collaboration with the NMEP arranged that trained entomologists and vector control researchers who benefited from the training programme be linked to their states malaria programmes, where each individual can develop proficiency and support the strategic plan of the national body to establish new sentinel sites for vector surveillance and insecticide monitoring in each state of the country.

## Conclusions

The capacity available for vector control research and surveillance at institutional level in Nigeria is weak. Increased training and access of personnel to relevant tools in malaria vector surveillance research is required in several institutions in all the six geopolitical zones. This will enhance the generation of a critical mass of well-trained scientists that can support local malaria vector control surveillance and research programmes.

## Additional files

**Additional file 1.** Pre and Post Test (Field).

**Additional file 2.** Pre and Post Test (Laboratory).
