# Supplementary material for: Outcome of capacity building intervention for malaria vector surveillance, control and research in Nigerian higher institutions
Source: Malar J. 2018 May 15;17:193. doi: 10.1186/s12936-018-2344-z (PMC5952629; doi:10.1186/s12936-018-2344-z)
Supplement: Supplementary file 1 — Additional file 1. Pre and Post Test (Field). [file 12936_2018_2344_MOESM1_ESM.docx]

| **S/N** | **CODE FOR PARTICIPANTS** | **Sex** | **Pre Test**  **%** | **Post Test**  **%** | **Difference** |
| --- | --- | --- | --- | --- | --- |
| **1** | AKAE | M | 39 | 61 | +22 |
| **2** | EJCE | M | 33 | 78 | +45 |
| **3** | FOOD | F | 11 | 71 | +60 |
| **4** | EDTE | M | 19 | 52 | +33 |
| **5** | CHAY | M | 28 | 65 | +37 |
| **6** | WAZS | M | 19 | 67 | +48 |
| **7** | SDAV | M | 10 | 52 | +42 |
| **8** | JPUQ | M | 29 | 79 | +50 |
| **9** | BARS | M | 17 | 71 | +54 |
| **10** | GNUP | M | 20 | 50 | +30 |
| **11** | ANOE | M | 37 | 72 | +35 |
| **12** | MANO | F | 25 | 83 | +58 |
| **13** | OLAO | F | 43 | 69 | +26 |
| **14** | ASAK | M | 22 | 69 | +47 |
| **15** | JABS | M | 38 | 79 | +41 |
| **16** | OOIF | M | 14 | 58 | +44 |
| **17** | SHNN | M | 43 | 82 | +39 |
| **18** | EAFG | M | 29 | 71 | +42 |
| **19** | EMCE | M | 47 | - | - |
| **20** | COAD | F | 17 | 61 | +44 |
| **21** | CBCU | F | 31 | 76 | +45 |
| **22** | ACIB | F | 32 | 69 | +37 |
| **23** | AUJA | M | 36 | 54 | +18 |
|  | **Overall Average** |  | 27.8 | 67.7 | +39.9 |
|  | **Male** |  | 28.2 | 66.3 | +38.1 |
|  | **Female** |  | 26.5 | 71.5 | +45 |
|  |  |  |  |  |  |

**^Additional file 1: Pre and Post – Intervention Test on Participants knowledge of field techniques in Malaria Vector Surveillance, Research and Control.^**

| t-Test: Paired Two Sample for Means | |  |
| --- | --- | --- |
|  |  |  |
|  | *Variable 1* | *Variable 2* |
| Mean | 27.7826087 | 67.82608696 |
| Variance | 115.9051383 | 96.4229249 |
| Observations | 23 | 23 |
| Pearson Correlation | 0.428733872 |  |
| Hypothesized Mean Difference | 0 |  |
| df | 22 |  |
| t Stat | -17.40950383 |  |
| P(T<=t) one-tail | 0.000000000000012 |  |
| t Critical one-tail | 1.717144374 |  |
| P(T<=t) two-tail | 0.0000000000000237 |  |
| t Critical two-tail | 2.073873068 |  |
