# Supplementary material for: Outcome of capacity building intervention for malaria vector surveillance, control and research in Nigerian higher institutions
Source: Malar J. 2018 May 15;17:193. doi: 10.1186/s12936-018-2344-z (PMC5952629; doi:10.1186/s12936-018-2344-z)
Supplement: Supplementary file 2 — Additional file 2. Pre and Post Test (Laboratory). [file 12936_2018_2344_MOESM2_ESM.docx]

| **S/N** | **CODE FOR PARTICIPANTS** | **Sex** | **Pre Test Percentage** | **Post Test Percentage** | **Difference** |
| --- | --- | --- | --- | --- | --- |
| 1. | A | M | 41 | 77 | +36 |
| 2. | B | M | 48 | 72 | +24 |
| 3. | C | M | 36 | 69 | +33 |
| 4. | D | M | 38 | 78 | +40 |
| 5. | E | M | 31 | 88 | +57 |
| 6. | F | M | 39 | 50 | +11 |
| 7. | G | M | 33 | 80 | +47 |
| 8. | H | F | 28 | 73 | +45 |
| 9 | I | M | 34 | 89 | +55 |
| 10. | J | F | 41 | 86 | +45 |
| 11. | K | F | 36 | 78 | +42 |
| 12. | L | F | 44 | 86 | +42 |
|  | Overall Average |  | 37.4 | 77.2 | +39.8 |
|  | Average Male Score |  | 37.5 | 75.4 | +37.9 |
|  | Average Female Score |  | 37.3 | 80.8 | +43.5 |

**Additional file 2: Pre and Post -Test Scores of Participants on Laboratory techniques in malaria vector control research**

| t-Test: Paired Two Sample for Means | |  |
| --- | --- | --- |
|  |  |  |
|  | *Variable 1* | *Variable 2* |
| Mean | 37.41666667 | 77.16667 |
| Variance | 31.71969697 | 115.6061 |
| Observations | 12 | 12 |
| Pearson Correlation | -0.121350896 |  |
| Hypothesized Mean Difference | 0 |  |
| df | 11 |  |
| t Stat | -10.8178209 |  |
| P(T<=t) one-tail | 0.00000016761 |  |
| t Critical one-tail | 1.795884819 |  |
| P(T<=t) two-tail | 0.00000033522 |  |
| t Critical two-tail | 2.20098516 |  |
